# Efficacy of mindfulness-based intervention for the treatment of chronic headaches: A systematic review and meta-analysis

**DOI:** 10.1016/j.amsu.2022.103862

**Published:** 2022-05-27

**Authors:** Muhammad Aemaz Ur Rehman, Radeyah Waseem, Ume Habiba, Muhammad Fahad Wasim, Soha Alam Rehmani, Maha Alam Rehmani, Maryam Abdullah, Yumna Khabir, Mahnoor Rehan Hashmi, Talal Almas, Syed Shahan Ali, Syed Muhammad Huzaifa Shah, Kaneez Fatima

**Affiliations:** a Department of Medicine, Dow University of Health Sciences, Karachi, Pakistan; b Department of Medicine, Baqai Medical University, Karachi, Pakistan; c Department of Medicine, Ziauddin University, Karachi, Pakistan; d Department of Medicine, RCSI University of Medicine and Health Sciences, Dublin, Ireland; e Department of Medicine, Liaquat College of Medicine and Dentistry, Karachi, Pakistan; f Department of Medicine, King Edward Medical University, Lahore, Pakistan

**Keywords:** Migraine, Tension-type headaches, Cluster headaches, Mindfulness-based stress reduction, Mindfulness-based cognitive therapy

## Abstract

**Background:**

Mindfulness-based stress reduction/cognitive therapy has attained popularity as an adjunctive treatment for a plethora of medical and psychiatric conditions, however, its impact on chronic headaches is inconclusive. This review aims to assess the impact of MBSR/MBCT in alleviating the symptoms of chronic headaches.

**Data sources and data selection:**

PubMed and Cochrane CENTRAL were searched from inception till 1st May 2021. Randomized Control Trials evaluating mindfulness-based stress reduction/cognitive therapy with either passive comparators (usual care) or active comparators (e.g., Health education or cognitive behavioral therapy) for chronic headaches (Migraine, Tension-type, or cluster headaches), which evaluated either headache frequency, pain intensity or headache duration as primary outcome were eligible for inclusion. The Risk of Bias was evaluated using the Cochrane Collaboration's Risk of Bias Tool.

**Results:**

A total of ten Randomized Controlled Trials (five on migraine; three on tension-type; two with mixed samples) were evaluated. In comparison to usual care, mindfulness-based stress reduction/cognitive therapy did not illustrate significant changes in headache frequency (MD = −0.14; 95% CI -1.26 to 0.97; P = 0.80; Moderate Certainty), headache duration (MD = −0.27; 95% CI -3.51 to 2.97, P = 0.87; Low Certainty) or pain intensity (MD = −0.19; 95% CI -0.46 to 0.07; P = 0.15; Moderate Certainty)

**Conclusion:**

The results found are insignificant for the three primary outcomes, which may be due to the low number of participants and often a high or unclear risk of bias in the randomized control trials included. Perhaps more aggressive clinical trials with a larger sample size effectively demonstrate differences in outcomes before and after therapy for MBSR/MBCT could provide a more significant change.

## Highlights

• Mindfulness-based therapy for treatment of chronic headache.
• Mindfulness vs usual care for treating patients with chronic headache.
• Effect of different mindfulness-based techniques over headache frequency, intensity and duration.
• Efficacy of Mindfulness-based therapy in achieving mindfulness in pts with chronic headache.

## 1 Introduction

Chronic Daily Headache (CDH) is a descriptive term rather than a single entity. It is commonly defined as headaches occurring on 15 or more days in a month for at least three months [1], as per the International Headache Society. The term amalgamates Tension-Type Headache, chronic migraine, and Chronic Cluster Headache. Generally, CDH affects around 1.7–4% of the world's adult population. The global estimation for the prevalence of a current headache disorder, which has been symptomatic at least once within the last year, is about 50% [2].

According to the Global Burden of Disease Study, headaches were collectively ranked as the third-highest cause of years lost due to disability (YLD) worldwide. Migraine was solely found to be the sixth highest cause [2]. Headaches are a global problem despite regional variations, affecting people of all age groups, races, income levels, and geographical areas. The condition has a debilitating effect on individuals and society through direct cost to healthcare and indirectly to the economy in general. Negative repercussions, both individually and socially, have been implicated. Direct financial prices due to the usage of the health care system, the indirect impact of sick leaves and reduced performances, and severance of relationship ties and family relations, along with reduced career opportunities and social rules, are to name. Still, a few harmful effects were noted [3].

Certain modifiable and non-modifiable risk factors may aggravate the development of chronic headaches. To name but a few, the modifiable factors such as sleep disorders, obesity, and high caffeine consumption may exacerbate the possibility of transforming episodic headaches into chronic headaches [4].

Many pharmacological treatments are available to minimize the functional disability caused by headaches. Despite the use of pharmacological treatment, many patients still suffer from a functional disability and pursue adjunctive therapies. Among these therapies, meditation-based mindfulness techniques have gained popularity in recent years. Mindfulness is a type of meditation that focuses on an intense awareness of sensations and being in the present, without interpretation or judgment. Practicing mindfulness involves breathing methods, guided imagery, and other practices to relax the body and mind and help reduce stress. Several clinical trials have been carried out to assess the effectiveness of mindfulness-based approaches for various chronic pain disorders. However, the evidence of their efficacy remains inconclusive for chronic headaches [5,6,7]. The purpose of our analysis was to determine the efficacy of Mindfulness-based Cognitive therapy (MBCT) and Mindfulness-based stress reduction (MBSR) in improving headache frequency, duration, and intensity in patients suffering from chronic headaches. Furthermore, we also assessed the differences in efficacy between different types of Mindfulness-based interventions (MBI) and the usual care.

## 2 Methods

This systematic review and meta-analysis was fully compliant with the preferred reporting items for the systematic review and meta-analyses (PRISMA) 2020 statement [8] and reports the required information accordingly. The compliance of our Meta was also assessed by the AMSTAR 2 guidelines [9].

### 2.1 Database and literature search strategy

We conducted the literature search using the following electronic databases: PubMed and Cochrane Central Register of Controlled Trials (The Cochrane Library) from inception till 1^st^ May 2021. The keywords used in our search string were “meditation” in combination with “Tension-type Headache” or “Tension Headache” or “TTH” or “Migraine with Aura” or “Migraine without Aura” or “migraine” or “Cluster headache” or “CH” or “Chronic Headache” or “Chronic daily headaches” or “Primary Headaches” or “hemicrania continua” or “HC” or “New daily persistent headache.” These searches were limited to English publications. All potentially relevant studies, articles (including undocumented data and meta‐analyses), and international guidelines were searched manually.

### 2.2 Selection procedure and eligibility criteria

The inclusion criteria were established as follows: (1) A randomized controlled trial; (2) included cluster headache or patients with tension-type headache and/or migraine; (3) compared MBSR or MBCT interventions to either a passive comparator (usual care) or an active comparator (e.g., Health education or cognitive behavioral therapy); (4) assessed headache frequency, duration and/or intensity as a primary outcome. Secondary outcomes of interest were mindfulness, safety, and patient adherence. The following exclusion criteria was applied: (a) observational studies, non-randomized trials, or pseudo-randomized trials, (b) tested interventions that differed clearly from the original MBSR/MBCT programs (e.g., acceptance and commitment therapy or dialectic behavioral therapy), and/or (c) had not been published as full-text articles in peer-reviewed scientific journals.

### 2.3 Data extraction and quality assessment

Studies were independently screened and assessed by two reviewers (RW and UH). The following data were obtained: study characteristics (e.g., author, year, and country); patient characteristics (e.g., age and sample size); description of interventions and duration, and outcomes measured. Two reviewers independently extracted these data using predefined criteria. Primary authors of the selected publications were contacted when the relevant information was not reported. In cases where a consensus could not be reached, the opinion of a third reviewer (FW) was sought.

### 2.4 Risk of bias assessment in individual studies

The quality of included studies was independently assessed by two authors (MAR, SAR), using the modified Cochrane Collaboration's risk of bias tool [10]. The risk of bias was judged as either low, unclear, or high risk in the following domains: Selection, performance, detection, attrition, reporting, and other biases. The original trial authors were contacted for further details if necessary. All analyses were based on previously published studies; thus, no ethical approval and patient consent were required.

### 2.5 Statistical analysis

All primary outcomes (Headache frequency, Headache duration, and Pain intensity) were analyzed using Mean Difference (MD), while the secondary outcome (Mindfulness) was calculated using Standard Mean Difference, and 95% CI. All values were calculated using RevMan by utilizing Pre-intervention and Post-intervention values. Where these were not cited, they were calculated from the standard errors, confidence intervals, or t values presented concerning headache frequency, duration, and pain intensity. A negative MD indicated beneficial effects for the MBSR/MBCT group when compared with the comparison group. For mindfulness, a positive SMD indicated beneficial effects for the mindfulness intervention. Heterogeneity was assessed by using I^2^ and Chi^2^ statistics (*P* < 0.10 was considered to be statistically significant) and was quantified by the *I*^*2*^ index (*I*^2^ >25%, >50%, and >75% indicate moderate, substantial, and considerable heterogeneity, respectively). Publication bias was not assessed due to the limited number of studies. A value of *P* < 0.05 was considered statistically significant. All statistical analyses were performed using Review Manager (RevMan) [Computer program]. Version 5.4, The Cochrane Collaboration, 2020 [11].

## 3 Results

**Fig. 1:**
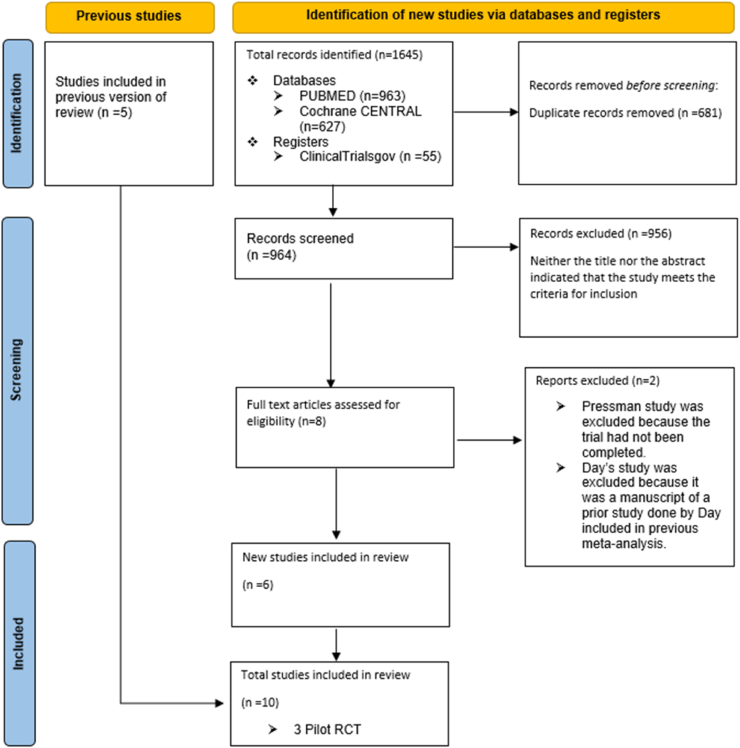
PRISMA

A total of 1645 articles were identified from the systematic literature search. After identification and elimination of duplicates using EndNote Reference Manager (Version X9; Clarivate Analytics, Philadelphia, Pennsylvania), 964 potentially relevant articles remained. Among those, 956 articles were eliminated during the title and abstract screening as they did not meet the inclusion criteria or were irrelevant. The remaining 8 articles were then subjected to full-text screening. Two articles were excluded after the full-text screening. Pressman et al., was excluded because the trial had not been completed [12] and Day et al., the study was excluded since it was a secondary analysis of an RCT that has already been included in our study [13]. 10 full-text articles (4 from previous meta-analysis and 6 recently published) were included in this meta-analysis and systematic review. One study from a previous meta-analysis [14] was not included since the data does not show the mean difference between the experimental and control group that was required in our analysis (Fig. 1).

### 3.1 Study characteristics

The characteristics of the ten RCTs included in this meta-analysis are summarized in Table 1. Of the ten RCTs, three were carried out in Iran [[5], [15], [16]], five in the United States of America [[6], [17], [18], [19], [20]], One in Australia [21], and one in Germany [7]. The mean age of all the patients included in the RCTs ranged between 18 and 65 years with a predominant population of females and had a diagnosis of chronic primary headache according to the International Classification of Headache Disorders criteria. Patients included in the study had been recruited from the local population [17,18,21,7] from local headache clinics [20], a university hospital [6,7], and a general hospital [[5], [15], [16]]. Two studies included patients identified with tension-type headache and migraine [16,18], two studies included patients with tension-type headache only [5,21], five studies included patients diagnosed with migraine only [17,[6], [19], [20],^7^] and one study included patients with primary headaches [15]. Four RCTs involved MBSR interventions that were adapted from the original MBSR program developed at the University of Massachusetts [5,17,6,7]. One study used an adaption of the mindfulness-based cognitive therapy (MBCT) program developed by Segal, Williams, and Teasdale [18] and one RCT used an adaption of the original MBSR program in combination with an adaption of the original MBCT program [21]. One study adapted the intervention from the MBCT for chronic headache pain protocol created by Day and colleagues [19] and one study followed *MBCT for chronic pain* from *A clinical manual and guide by* John Wiley Sons [15]. One study involved the enhanced MBSR (MBSR1) program that included 8 weekly sessions, adapting the original MBSR program developed by Kabat-Zinn, followed by 4 biweekly sessions of enhanced meditation techniques [20]. In one study MBSR treatment was performed as bibliotherapy based on an 8-week treatment protocol [16]. Only four of the included RCTs reported on funding [15,[6], [17], [18]]. Three studies were sponsored by grants from organizations that have no associations with the trials. One study was supported by the Anthony Marchionne Foundation and the National Headache Foundation [18]. One study received a grant from the 10.13039/100014832American Headache Society and the Research Fund of the John Graham Headache Center [17] and the other study had financial support from the Islamic Azad University of Alborz [15] and one study was supported by the National Institute of Health [6].

**Table 1:** Characteristics of the included studies.

Author, Year	No. Of Participant s, No. Of Groups	Mean Age	Inclusion criteria	Treatment group: Intervention, Program length, frequency,	Control group: Intervention, Program length, frequency, duration.	Post treatment assessment points	Outcome measures	Results.
[6] Wells, 2021	89,2	44(TG), 44(CG)	Diagnosis of migraine according to ICHD-II criteria, 4–20 migraine days/month, > 1 year history of migraines, >18 years old.	MBSR Program for 2 h per week for 8 weeks, retreat day(optional). Homework: 30 min per day.	No MBSR sessions were given, participants continued usual care (Headache education).	Post intervention: after 12 weeks of intervention. Follow-up: at week 24 and then at week 36.	Primary outcome: 1. Change in monthly migraine day frequency. Secondary outcomes: 2. Frequency 3. Intensity 4. Unpleasantness 5. Duration 6. Disability 7. Quality of life 8. Wellbeing measures.	Significant group differences in: 6. Disability 7. Quality of life 8. Well-being measures.
[19] Seng, 2019	60,2	36.2(TG) 44.2(CG)	Diagnosis of migraine according to ICHD-III criteria, >6 headache days/month,18–65 years of age.	MBSR Program for 2 h per week for 8 weeks, retreat day(optional). Homework: 30	No MBCT sessions were given, participants continued wait listing and treatment as usual.	Post intervention: after 30 days post treatment. Follow-ups: at month 2 and then at month 4.	Primary outcome: I. Disability. Secondary outcomes: 2. Frequency(days/month). 3.Intensity.	Significant group differences in I. Disability.
[16] Tavallaei, 2018	30,2	32.4(TG) 34.8(CG)	Diagnosis of tension type headache and migraine headache according to IAH-2, 18–50 years of age, least education degree of diploma.	MBSR program for 30 min per week for 8 weeks. Homework: Frequency and duration not mentioned	No MBSR sessions were given, participants continued usual care.	Post intervention: after 8 weeks of intervention. Follow up: No long-term assessment point	Primary outcome: 1 Pain intensity 2 Distress 3 Disability 4 Mindfulness.	Significant group differences in: 1 Pain intensity 2 Distress 3 Disability 4 Mindfulness.
[15] Namjoo, 2019	85,2	36.7(TG) 38.2(CG)	Diagnosis of primary headache according to the ICHD-III criteria, at least 3 days per month for more than 3 months, 19 years of Age or Older	MBCT program for 2 h per week for 8 weeks. Homework: Frequency and duration not mentioned.	No MBCT sessions were given, participants received attention and therapist's empathy and participated in group discussion.	Post intervention: after 8 weeks of intervention. Follow up: 3 months after intervention.	Primary outcome: 1 Pain interference (BPI scale), 2. Pain severity (lOpoint NRS). Secondary outcomes: 3. Pain diversion, 4. Pain focus, 5. Pain distancing, 6. Pain openness (5-point Likert scale).	Significant group differences in: 4. Pain focus 6. Pain openness.
[20] Seminowicz, 2020	98,2	42.3(TG), 43.0(CG)	More than 12 months diagnosis of migraine headache with or without aura according to the ICHD criteria, at least 4–14 headaches in 28 days,18–65 years of age.	12 MBSR + sessions of 2 h per week for 8 weeks then biweekly for another 8weeks, Homework: Frequency and duration not mentioned.	SMH of 12 sessions for 4 months	Post intervention: after 10 weeks of intervention. Follow up: at week 20 and then at week 52.	Primary clinical outcome: 1 Frequency (headache diary days/28days). Primary imaging outcome: 2 Brain activation during cognitive task. Secondary clinical outcomes: 3 Disability 4 Intensity 5 Response to Rx. Secondary imaging outcome: 6 Cognitive efficiency.	Significant group differences in: 1 Frequency 3 Disability 5 Response to RX 6 Cognitive efficiency.
[7] Simshauser, 2019	61,2	43.8(TG), 43.9(CG)	Diagnosis of migraine with or without aura according to the ICD-10 criteria, at least 2 attacks per month, 18–65 years of Age.	MBSR Program for 2.5 h per week for 8 weeks, Homework: Frequency and duration not mentioned. In addition to the regular sessions, a 6-h silent retreat was held during the 6th week of the course	Education and relaxation program for 2.5 h 3 times within 8 weeks.	Post intervention: after 8 weeks of intervention. Follow up: 3 months after intervention.	Primary outcome: 1 Frequency (headache diary days/months). Secondary outcomes: 2. Pain- related impairment (4point scale), 3. Frequency of rescue medication use (days/month), 4. Psychological variables.	Significant group differences in: 4 Psychological variables.
[18] Day, 2014	34,2	43.1(TG), 40.1(CG)	Diagnosis of primary headache according to the ICHS-II, 3 or more days per month for more than 3 months, Headache pain the primary source of pain, if using headache medications must have begun at least 4 weeks before baseline assessment, reading ability was sufficient to comprehend self-monitoring form, 19 years of Age or Older.	MBCT program plus TAU for 2 h per week for 8 weeks. Homework: daily practice meditation 45 min for 6 days per week	No MBCT sessions were given, participants continued usual care.	Post intervention: after 8 weeks of intervention. Follow up: No long term assessment point	Primary outcomes: 1 Frequency (headache diary. 2. Duration (headache diary; minutes). 3. Intensity (headache diaiy; 0–10 VAS). Secondary outcomes: 4 Mindfulness (headache diary; 1–6 Likert scale).	No significant group differences.
[19] Cathcart, 2014	32,2	45.8(TG); 45.3(CG)	Diagnosis of tension type headache according to the ICHD-II criteria, 18–65 years of age, no other headache or pain symptoms, including suspected or probable Medication Overuse Headache.	MBT program based on MBSR for 2 h twice weekly for 3 weeks. Homework: daily, 30 min.	No MBT sessions were given, participants continued usual care.	Post intervention: after 3 weeks of intervention. Follow up: No long term assessment point.	1 Frequency (headache diary; days/fortnight). 2.Duration (headache diary; hours). 3.Intensity (headache diary; 6-point NRS). 4.Mindfullness (FFMQ) no primary outcome defined.	Significant group differences in I. Frequency.
[5] Omidi, 2014	60,2	34.5(TG), 32.0(CG)	Diagnosis of tension type headache according to the ICHD-II criteria, >18 years of age.	MBSR Program for 2 h per week for 8 weeks, Homework: Frequency and duration not mentioned.	No MBSR sessions were given, participants continued usual care.	Post intervention: after 8 weeks of intervention. Follow up: 3 months after intervention.	I. Intensity (headache diary: 11 point NRS, 2 Mindfulness (MAAS) no primary outcome defined.	Significant group differences in: 1 Intensity 2 Mindfulness.
[17] Wells, 2014	19,2	45.9(TG), 45.2(CG)	Diagnosis of migraine with or without Aura according to ICHD-II, 4–14 migraine days/month, > 1 year history of migraines, >18 years old.	MBSR Program for 2 h per week for 8 weeks, retreat day (6 h), Homework: 45 min for at least 5 days per week. Yoga was the part of the curriculum.	No MBSR sessions were given, participants continued usual care.	Post intervention: after 28 days after intervention. Follow up: No long term assessment point.	Primary outcome: 1 Frequency (headache diary days/month). Secondary outcomes: 2 Duration (headache diary; hours), 3 Intensity (headache diary 11- point NRS), 4 Mindfulness (FFMQ).	Significant group differences in: 4 Mindfulness.

### 3.2 Risk of bias assessment

The risk of bias for each included RCT is shown in Table 2. Of the 10 eligible studies, five of them stated adequate random sequence generation and allocation concealment (selection bias) [17,[6], [19], [20],^7^], the randomization process in two studies was not explained [5,21], Three studies did not report an adequate form of allocation concealment [[5], [15], [16]]. Two studies attempted to ‘blind’ their participants to treatment allocation. One did by informing participants that the MBSR course will have two start times, with randomization to either date [17], and one recruited participants by describing the study as sessions where they will attain knowledge that may help headaches without medications with course material unrevealed [17]. Four studies gave insufficient information regarding the blinding of participants and personnel and hence judged as unclear [5,16,21,7] and for three studies, the risk of performance bias was judged as high [15,18,19]. Three studies reported ‘blinding’ of outcome assessments [6,20,21]. The risk of attrition bias was deemed high in two RCTs [15,21] and the risk of reporting bias was found high in one of the included studies [5] whereas the selection bias, performance bias, detection, bias and other forms of bias were judged as high risk in one study [18]. Explanations for each judgment are specified in a table provided as supplemental material (Supplemental Figs. 2a and 2b).

**Table 2:** Summary of quality assessment.

Author, year Bias	Random sequence generation (selection bias)	Allocation concealment (selection bias)	Blinding of participants and personnel (performance bias)	Blinding of outcome assessment (detection bias)	Incomplete outcome data (attrition bias)	Selective reporting (reporting bias)	Other Bias
[18] Wells, 2021	Low risk	Low risk	Low risk	Low risk	Low Risk	Low Risk	Low risk
[6] Seng, 2019	Low risk	Low risk	High risk	High risk	Low risk	Low risk	Low risk
[15] Tavallaei, 2018	Low risk	Unclear	Unclear	Unclear	Low risk	Low risk	Low risk
[5] Namjoo, 2019	Low risk	Unclear	High risk	High risk	High risk	Low risk	Low risk
[19] Seminowicz, 2020	Low risk	Low risk	Unclear^a^/Low risk^b^	Low risk	Unclear	Low risk	Low risk
[21] Simshauser, 2019	Low risk	Low risk	Unclear	Unclear	Low risk	Low risk	Low risk
[17] Day, 2014	High risk	High risk	High risk	High risk	Low risk	Low risk	High risk
[20] Cathcart, 2014	Unclear	Low risk	Unclear	Low risk	High risk	Low risk	Low risk
[14] Omidi, 2014	Unclear	Unclear	Unclear	Unclear	Low risk	High risk	Low risk
[16] Wells, 2014	Low risk	Low risk	Low risk^c^/Unclear^d^	Unclear	Low risk	Low risk	Low risk

a Blinding of participants not reported.

b Therapists blinded.

c Attempt to blind patients.

d Therapists not blinded

### 3.3 Results of meta-analysis

**Fig. 2:**
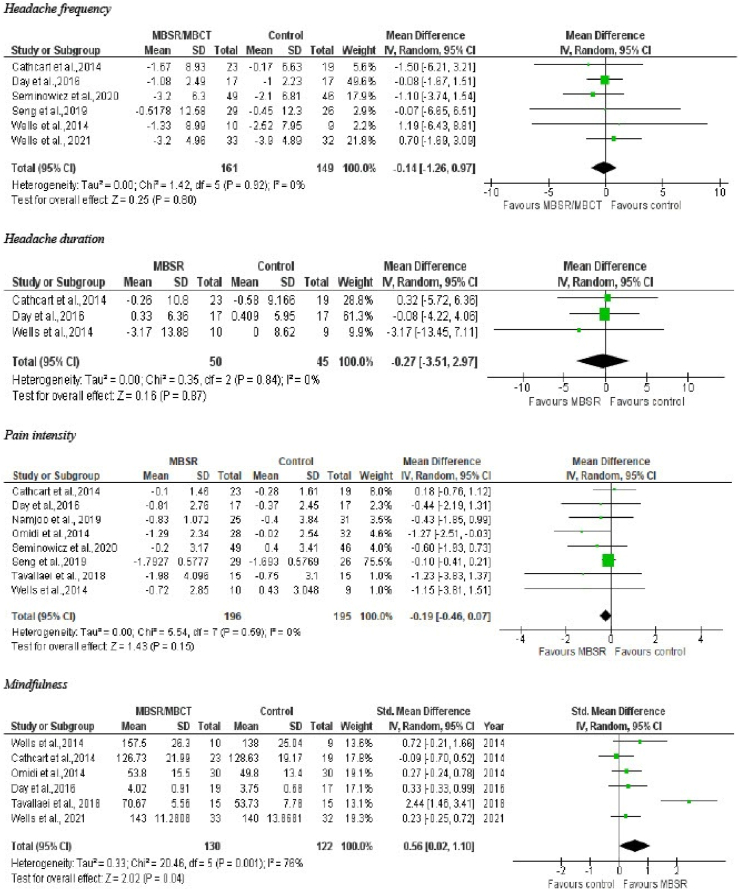
Meta-analysis headache frequency, headache duration, pain intensity, mindfulness. CI: Confidence Interval; SD: Standard Deviation; MBSR: Mindfulness Based Stress Reduction; MBCT: Mindfulness Based Cognitive Therapy.

The summarized results of our meta-analysis are presented in Fig. 2.

### 3.4 Effects on primary outcomes

#### 3.4.1 Headache frequency

Headache frequency was the reported outcome in six included studies in our meta-analysis. The combined headache frequency response was (MD = - 0.14; 95% CI -1.26 to 0.97; P = 0.80; Moderate Certainty) with heterogeneity (I^2^ = 0%) showing MBSR/MBCT had no significant improvement in headache frequency compared to usual care.

#### 3.4.2 Headache duration

Three RCTs included headache duration as their reported outcome. Compared to usual care, MBSR/MBCT was not associated with a statistically significant improvement in Headache duration (MD = -0.27; 95% CI -3.51 to 2.97; P = 0.87; Low Certainty) with heterogeneity (I^2^ = 0%).

#### 3.4.3 Pain intensity

Eight included studies reported pain intensity as their outcome. The combined pain intensity response was (MD = - 0.19; 95% CI -0.46 to 0.07; p = 0.15; Moderate Certainty) with heterogeneity (I^2^ = 0%) showing MBSR/MBCT had insignificant effect in headache pain intensity compared to usual care.

#### 3.4.4 Effects on secondary outcomes

In reference to the secondary outcome of Mindfulness, the difference between MBSR/MBCT and control groups was shown to feature a statistically significant difference (six RCTs; SMD = 0.56; 95% CI 0.02 to 1.10; P = 0.04; Moderate Certainty). The values are obtained by comparison between post-treatment values for MBSR/MBCT and control, both.

### 3.5 Patient safety

Of the 10 included trials, three reported the incidence of adverse events [[6], [18], [19]], six RCTs did not report the occurrence (or absence) of adverse events [[5], [7], [15], [16], [18], [21], [22]], while one RCT described that no adverse events had occurred and that no patients withdrew from the study as a result [17] and two studies did not mention any reasons for patient withdrawal (i.e., ‘drop-outs’) [5,21].

## 4 Discussion

The results of this systematic review provide limited evidence that MBSR/MBCT intervention may be effective in reducing the frequency, duration, and pain intensity of headaches, in patients with chronic headaches including tension-type and migraine compared with the usual care but it demonstrated a significant difference between groups in achieving mindfulness. We used the mean difference (pre-intervention – post-intervention) for MBSR/MBCT and control respectively. To get this mean difference, the generic inverse variance was first used followed by the continuous data option to compare them which gave the final forest plot. In studies where more than one follow-up was present and all values showed a significant change, we used the values of the last follow up but if the results stopped showing significant changes till the last follow-up, we used the most significant value. Despite statistically low heterogeneity, no strong conclusion could be drawn about the efficacy of MBSR/MBCT as the confidence intervals for headache frequency, headache duration, and pain intensity containing medium or high effect sizes both in favor of MBSR/MBCT and in favor of usual care. The analysis using the DerSimonianLaird estimator found no statistically significant difference between groups. One study indicated a reduction in headache duration in patients with episodic migraine after Enhanced mindfulness-based stress reduction treatment. The available data on treatment adherence and patient safety was also inconclusive. Although few studies indicated that MBSR/MBCT may be well tolerated, there was inadequate evidence to draw any firm conclusions about the safety of this therapy.

This review is an update to the recent meta-analysis as it includes new studies with larger population sizes. The updated findings partly met with the results of a previous meta-analysis of mindfulness-based stress reduction for treating chronic headaches. The review found the differences between MBSR/MBCT and usual care in improving headache frequency, duration, and pain intensity to be insignificant. The analysis like the current review, also found MBSR/MBCT to be no more effective than usual pharmacological interventions. Since the last analysis unlike the current evidence showed insignificant results on the level of mindfulness achieved the findings are only partially comparable.

While this review was systematic and comprehensive, the findings of this study have to be seen in the light of some limitations. The primary limitation is an inadequate sample size of all the included studies which may have introduced selection bias. Although the number of participants has increased in RCTs following the previous meta-analysis, it is still inconsequential. It's suggested that the trials examining the effectiveness of MBCT/MBSR are conducted over a larger sample size selected from different health centers to increase generalizability and generate more inclusive conclusions.

The second limitation concerns the fact that overall, the risk of bias in included studies was unclear for many domains. Only Wells et al., study 2021 has low risk in all domains. However, most of the included studies following the previous meta-analysis have a low risk of bias in the most important domains especially selection and reporting bias. These potential biases should be taken into account when interpreting the findings of this review. Thirdly, the mean age of all the patients included in the RCTs ranged between 18 and 65 years with a predominant population of females which limits the generalizability of the findings to people with chronic headaches who are men or people over the age of 65. Another major limitation is that many of the considered studies used paper versions of health diaries which may have contributed to false-positive findings hence the administration of electronic diaries can be useful in improving the accuracy of results. The studies did not investigate the dose-response pattern which would have contributed to imprecise results as different age groups may respond to similar doses differently. It is suggested that future studies perform dose-response analysis to determine the optimal amount of MBCT/MBSR. High dropout values demonstrated the need for intention-to-treat analyses. Future research comparing intervention groups to the active control group is required. Longer follow-up periods are necessary to reduce baseline variability. Other limitations include differences in MSBR/MBCT interventions in various studies as well as a difference in the follow-up period. This together with the attrition [5,20] and reporting bias [14] may have influenced the results.

## 5 Conclusion

This is an updated systematic review and meta-analysis, with a specific focus on the effectiveness and safety of MBCT/MBSR for patients with all types of chronic headaches. Like the preceding ones, it found no evidence that MBSR/MBCT is effective in improving the frequency, duration, and intensity of headaches but showed considerable improvement in mindfulness achieved in patients suffering from chronic headaches. The minimal number of RCTs included in this review, the low total number of participants in each included study, and the high/unclear risk of bias in included trials all contributed to the imprecise findings of this review. Given these findings, the use of MBSR/MBCT interventions for the treatment of migraine and/or tension-type headaches cannot be recommended at this time but it can be considered as a possible option for the management of chronic headaches as a stand-alone treatment or along with usual medications in some specific situations. For building more concrete evidence and making a clearer recommendation, future investigations with the aforementioned implications need to be conducted.

## Sources of funding

None to declare

## Consent to participate

Not applicable.

## Consent for publication

Not applicable.

## Provenance and peer review

Not commissioned, externally peer-reviewed.

## Ethical Approval

N/A.

## Consent

N/A.

## Author contribution

Talal Almas - Conceptualization and Designing Study

Aemaz Ur Rehman– Intro and Methods (Manuscript Writing), Data Collection,

Radeyah Waseem – Into and Methods (Manuscript writing), Data Collection, Baseline Characteristics, Forest Plots, Manuscript editing, formatting

Ume Habiba – Into and Methods (Manuscript writing), Data Collection, Baseline Characteristics, Forest Plots Manuscript editing, formatting

Muhammad Fahad Wasim – Forest Plots, Data Collection, Manuscript editing, formatting

Soha Alam Rehmani– Manuscript writing (Abstract, Discussion, Limitations, Conclusion), Data Collection

Maha Alam Rehmani- Manuscript writing (Abstract, Discussion, Limitations, Conclusion), Data Collection

Maryam Abdullah – Forest Plots, Data Collection

Yumna Khabir- Forest Plots, Data Collection

Syed Shahan Ali – Manuscript editing, Data Collection

Syed Muhammad Huzaifa Shah - Forest Plots , Data Collection

Kaneez Fatima - Conceptualization and Designing Study

## Registration of Research Studies

1. Name of the registry:
2. Unique Identifying number or registration ID:
3. Hyperlink to your specific registration (must be publicly accessible and will be checked):

## Guarantor

Kaneez Fatima.

kaneezfatima344@gmail.com.

## Declaration of competing interest

The authors declare that they have no conflict of interest.
