# Supplementary material for: Efficacy of mindfulness-based intervention for the treatment of chronic headaches: A systematic review and meta-analysis
Source: Ann Med Surg (Lond). 2022 May 27;78:103862. doi: 10.1016/j.amsu.2022.103862 (PMC9207091; doi:10.1016/j.amsu.2022.103862)
Supplement: Multimedia component 1 [file mmc1.docx]

SUPPLEMENTARY MATERIAL

| Database  (Articles  Retrieved) | Search Strategy. |
| --- | --- |
|  | ("MBSR"[All Fields] OR "MBCT"[All Fields] OR ("mind s"[All Fields] OR "minded"[All Fields] OR "mindful"[All Fields] OR "mindfulness"[MeSH Terms] OR "mindfulness"[All Fields] OR "minding"[All Fields] OR "minds"[All Fields]) OR ("mind s"[All Fields] OR "minded"[All Fields] OR "mindful"[All Fields] OR "mindfulness"[MeSH Terms] OR "mindfulness"[All Fields] OR "minding"[All Fields] OR "minds"[All Fields]) OR ("meditate"[All Fields] OR "meditated"[All Fields] OR "meditating"[All Fields] OR "meditation"[MeSH Terms] OR "meditation"[All Fields] OR "meditations"[All Fields] OR "meditational"[All Fields] OR "meditative"[All Fields] OR "meditator"[All Fields] OR "meditators"[All Fields])) AND ("tension type headache"[MeSH Terms] OR ("tension type"[All Fields] AND "headache"[All Fields]) OR "tension type headache"[All Fields] OR ("tension"[All Fields] AND "type"[All Fields] AND "headache"[All Fields]) OR "tension type headache"[All Fields] OR ("tension type headache"[MeSH Terms] OR ("tension type"[All Fields] AND "headache"[All Fields]) OR "tension type headache"[All Fields] OR ("tension"[All Fields] AND "headache"[All Fields]) OR "tension headache"[All Fields]) OR "TTH"[All Fields] OR ("migraine with aura"[MeSH Terms] OR ("migraine"[All Fields] AND "aura"[All Fields]) OR "migraine with aura"[All Fields]) OR ("migraine without aura"[MeSH Terms] OR ("migraine"[All Fields] AND "aura"[All Fields]) OR "migraine without aura"[All Fields]) OR ("migrain"[All Fields] OR "migraine disorders"[MeSH Terms] OR ("migraine"[All Fields] AND "disorders"[All Fields]) OR "migraine disorders"[All Fields] OR "migraine"[All Fields] OR "migraines"[All Fields] OR "migraine s"[All Fields] OR "migraineous"[All Fields] OR "migrainers"[All Fields] OR "migrainous"[All Fields]) OR ("cluster headache"[MeSH Terms] OR ("cluster"[All Fields] AND "headache"[All Fields]) OR "cluster headache"[All Fields]) OR ("chemistry"[MeSH Subheading] OR "chemistry"[All Fields] OR "ch"[All Fields]) OR ("headache disorders"[MeSH Terms] OR ("headache"[All Fields] AND "disorders"[All Fields]) OR "headache disorders"[All Fields] OR ("chronic"[All Fields] AND "headache"[All Fields]) OR "chronic headache"[All Fields]) OR ("headache disorders"[MeSH Terms] OR ("headache"[All Fields] AND "disorders"[All Fields]) OR "headache disorders"[All Fields] OR ("chronic"[All Fields] AND "daily"[All Fields] AND "headaches"[All Fields]) OR "chronic daily headaches"[All Fields]) OR (("primaries"[All Fields] OR "primary"[All Fields]) AND ("headache"[MeSH Terms] OR "headache"[All Fields] OR "headaches"[All Fields] OR "headache s"[All Fields])) OR (("headache"[MeSH Terms] OR "headache"[All Fields] OR "hemicrania"[All Fields] OR "hemicranias"[All Fields]) AND "continua"[All Fields]) OR ("rev hosp clin fac med sao paulo"[Journal] OR "hum comput fairfax"[Journal] OR "hc"[All Fields]) OR ("New"[All Fields] AND ("dailies"[All Fields] OR "daily"[All Fields]) AND ("persist"[All Fields] OR "persistance"[All Fields] OR "persistant"[All Fields] OR "persisted"[All Fields] OR "persistence"[All Fields] OR "persistences"[All Fields] OR "persistencies"[All Fields] OR "persistency"[All Fields] OR "persistent"[All Fields] OR "persistently"[All Fields] OR "persistents "[All Fields] OR "persister"[All Fields] OR "persisters"[All Fields] OR "persisting"[All Fields] OR "persists"[All Fields]) AND ("headache"[MeSH Terms] OR "headache"[All Fields] OR "headaches"[All Fields] OR "headache s"[All Fields]))) |
|  |  |

Supplemental Figure 1. Search Strategy used in the database searched

Figure 2a. Quality Assessment of Included Randomized Controlled Trials using Cochrane Risk of Bias Tool; Risk of Bias Graph


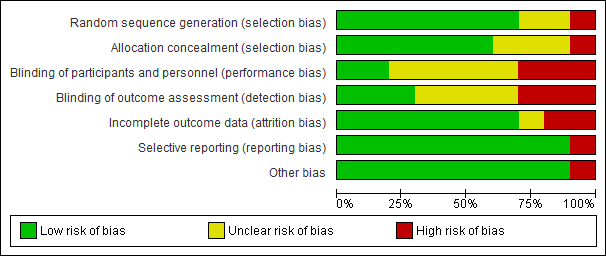


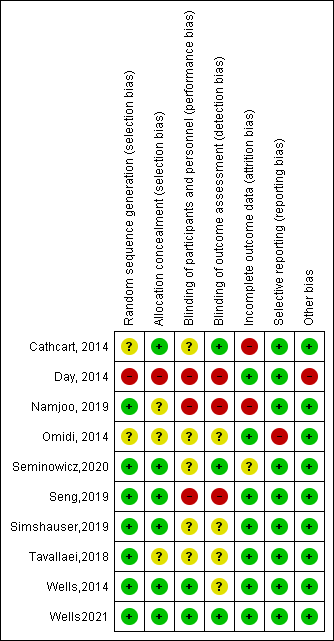


Figure 2b. Quality Assessment of Included Randomised Controlled Trials using Cochrane Risk of Bias Tool; Risk of Bias Summary


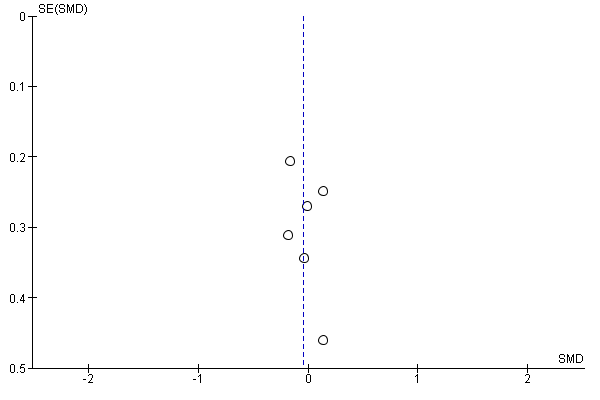


Figure 3. Forest Plot for headcahe freuqency


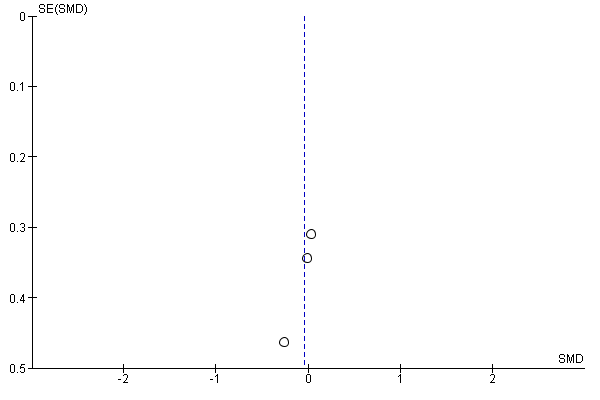


Figure 4. Forest Plot for Headache duration


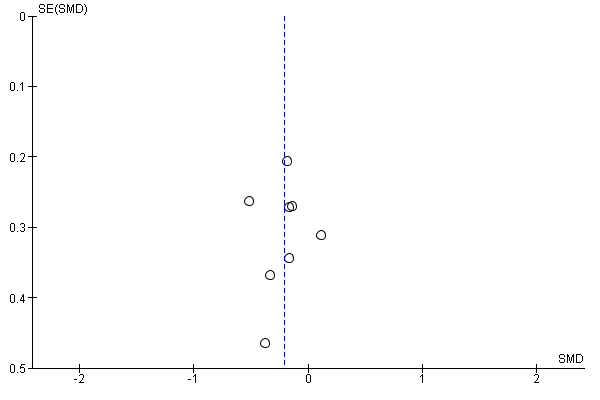


Figure 5. Forest Plot for Pain Intensity


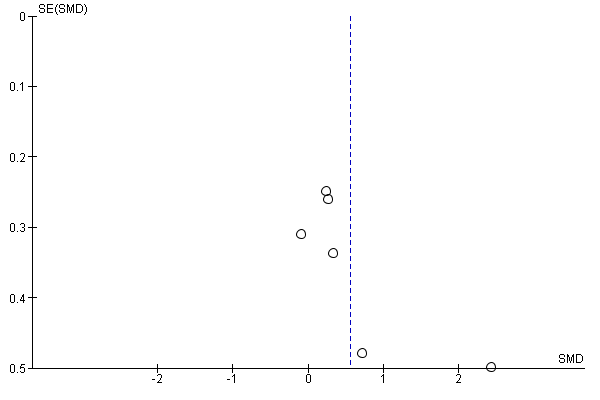


Figure 6. Forest Plot for Mindfulness
